# Jmjd2c facilitates the assembly of essential enhancer-protein complexes at the onset of embryonic stem cell differentiation

**DOI:** 10.1242/dev.142489

**Published:** 2017-02-15

**Authors:** Rute A. Tomaz, Jennifer L. Harman, Donja Karimlou, Lauren Weavers, Lauriane Fritsch, Tony Bou-Kheir, Emma Bell, Ignacio del Valle Torres, Kathy K. Niakan, Cynthia Fisher, Onkar Joshi, Hendrik G. Stunnenberg, Edward Curry, Slimane Ait-Si-Ali, Helle F. Jørgensen, Véronique Azuara

**Affiliations:** 1 Institute of Reproductive and Developmental Biology, Faculty of Medicine, Imperial College London, London W12 0NN, UK; 2 Cardiovascular Medicine Division, Department of Medicine, University of Cambridge, Cambridge CB2 0QQ, UK; 3 Centre National de la Recherche Scientifique CNRS - Université Paris Diderot, Sorbonne Paris Cité, Epigenetics and Cell Fate, UMR 7216 CNRS, Paris 75013, France; 4 Human Embryo and Stem Cell Laboratory, The Francis Crick Institute, London NW7 1AA, UK; 5 Wellcome Trust Sanger Institute, Hinxton, Cambridge CB10 1HH, UK; 6 Radboud University, Faculty of Science, Department of Molecular Biology, Nijmegen 6525GA, The Netherlands

**Keywords:** Jmjd2c (Kdm4c), Enhancers, Gene regulation, Embryonic stem cells, Epiblast stem cells, Lineage specification

## Abstract

Jmjd2 H3K9 demethylases cooperate in promoting mouse embryonic stem cell (ESC) identity. However, little is known about their importance at the exit of ESC pluripotency. Here, we reveal that Jmjd2c facilitates this process by stabilising the assembly of mediator-cohesin complexes at lineage-specific enhancers. Functionally, we show that Jmjd2c is required in ESCs to initiate appropriate gene expression programs upon somatic multi-lineage differentiation. In the absence of Jmjd2c, differentiation is stalled at an early post-implantation epiblast-like stage, while *Jmjd2c*-knockout ESCs remain capable of forming extra-embryonic endoderm derivatives. Dissection of the underlying molecular basis revealed that Jmjd2c is re-distributed to lineage-specific enhancers during ESC priming for differentiation. Interestingly, Jmjd2c-bound enhancers are co-occupied by the H3K9-methyltransferase G9a (also known as Ehmt2), independently of its H3K9-modifying activity. Loss of Jmjd2c abrogates G9a recruitment and further destabilises loading of the mediator and cohesin components Med1 and Smc1a at newly activated and poised enhancers in ESC-derived epiblast-like cells. These findings unveil Jmjd2c and G9a as novel enhancer-associated factors, and implicate Jmjd2c as a molecular scaffold for the assembly of essential enhancer-protein complexes with an impact on timely gene activation.

## Abstract (precis)

**Highlighted article:** Jmjd2c is required for the proper assembly of mediator-cohesin complexes at lineage-specific enhancer regions and is essential for timely gene activation.

## INTRODUCTION

Pluripotency, the ability of a cell to generate all somatic lineages, is transiently acquired *in vivo* during mammalian pre-implantation development. Upon blastocyst formation, pluripotent cells develop within the inner cell mass (ICM), a mosaic of cells surrounded by an extra-embryonic layer – the trophectoderm. By the time of implantation, a second extra-embryonic lineage, the primitive endoderm, emerges at the ICM surface. Concurrently, the ICM maintains its pluripotency as it matures into the epiblast but ultimately goes on to form the three primary germ layers and germ cells upon gastrulation (Boroviak and Nichols, 2014; Rossant, 2008).

Pluripotent mouse embryonic stem cells (ESCs) are derived from ICM cells, and can self-renew and faithfully maintain an undifferentiated state *in vitro* in the presence of leukaemia inhibitory factor (LIF) and serum components, while preserving their multi-lineage differentiation capacity (Evans and Kaufman, 1981; Martin, 1981; Niwa et al., 1998; Ying et al., 2003). Most recently, stem cell lines with similar lineage potential were established from other developmental stages (Chung et al., 2006; Tesar, 2005), including a number of post-implantation epiblast-derived stem cells (EpiSCs) (Brons et al., 2007; Osorno et al., 2012; Tesar et al., 2007). While ESCs are thought to represent an immature (pre-implantation) phase of pluripotency, EpiSCs exist in a more advanced state on the verge of differentiation (Nichols and Smith, 2009). Moreover, ESCs can stably transit into self-renewing EpiSCs, acquiring characteristics of post-implantation epiblast-like cells (Guo et al., 2009).

ESC abilities depend on the potent expression of self-renewal genes and transcriptional priming of silent, lineage-affiliated genes – a crucial balance of gene expression maintained through crosstalk between transcriptional factors and chromatin regulators (Azuara et al., 2006; Bernstein et al., 2006; Chen and Dent, 2014; Ng and Surani, 2011; Stock et al., 2007). Remarkably, both active (ESC-specific) and primed (lineage-specific) genes are expressed in a heterogeneous manner, a feature long considered to be a hallmark of ESC cultures that safeguards the swift response to differentiation cues (Efroni et al., 2008; Torres-Padilla and Chambers, 2014). Yet, it is now possible to derive and maintain ESCs with reduced heterogeneity and transcriptional gene priming through chemical inhibition of two differentiation-associated pathways, Mek and Gsk3 (2i conditions), capturing a naïve pluripotent state *in vitro* (Marks et al., 2012; Ying et al., 2008).

Gene promoter regions enriched in CpG islands and H3K4me3 function as genomic platforms for the recruitment of transcription factors and co-regulators, as well as for the basal transcriptional machinery (Deaton and Bird, 2011; Illingworth and Bird, 2009). Moreover, distal DNA elements such as enhancers play a significant role in potentiating gene expression being typically decorated by H3K4me1 and bound by pioneer transcription factors (Calo and Wysocka, 2013; Gibcus and Dekker, 2013; Spitz and Furlong, 2012). For example, the core pluripotency factor Oct4 was commonly shown to mark both active and poised enhancers in ESCs and EpiSCs (Buecker et al., 2014; Calo and Wysocka, 2013). Enhancer activity and robust ESC-specific gene expression entail long-range DNA interactions with the transcriptional apparatus at promoters, involving the cooperative action of mediator-cohesin complexes (Kagey et al., 2010). Yet, relatively little is known about the identity of proteins that stabilise the formation of such assemblies.

Histone demethylases have emerged as key players in the control of cell identity and development, mainly through modulation of the chromatin environment of tissue-specific genes (Nottke et al., 2009). Recently, additional roles for these molecules independent of their enzymatic activity have been reported (Shpargel et al., 2012; Wang et al., 2012; Yang et al., 2010), especially in regulating the recruitment of Polycomb repressive complexes (PRC) and poised RNA polymerase II to the promoter regions of developmental genes in ESCs (Farcas et al., 2012; Wu et al., 2013). Jmjd2c (also known as Kdm4c) is a member of the Jmjd2 gene family initially identified as H3K9me2/3 and/or H3K36me2/3 histone demethylases (Chen et al., 2006; Klose et al., 2006; Whetstine et al., 2006). Jmjd2c is highly expressed in the early embryo and in ESCs (Boroviak et al., 2015; Burton et al., 2013; Loh et al., 2007; Wang et al., 2010), and RNA interference-mediated depletion of the protein was shown to impair cleavage-stage development and ESC integrity, as well as inhibiting somatic cell reprogramming (Das et al., 2014; Loh et al., 2007; Wang et al., 2010). *Jmjd2c*-null ESCs and mice could, however, be generated via gene trap approaches (Pedersen et al., 2014), in agreement with functional redundancy between *Jmjd2* gene family members to support cell proliferation and survival (Pedersen et al., 2016). At the genomic level, Jmjd2c preferentially targets H3K4me3-rich promoter regions of active and development-associated genes in ESCs via its Tudor domains (Das et al., 2014; Pedersen et al., 2014), where Jmjd2c is proposed to assist Jmjd2b-Nanog and PRC2 in transcriptional activation and repression, respectively (Das et al., 2014).

In this study, we uncover a previously unrecognised link between Jmjd2c recruitment to lineage-specific enhancers and the establishment of a functionally primed state for differentiation *in vitro*. We show that, in the absence of Jmjd2c, ESC differentiation is severely impeded at an early post-implantation epiblast-like stage. Although *Jmjd2c*-knockout ESCs can transit into self-renewing EpiSCs, these cells fail to form derivatives of the three primary germ layers, as revealed by their inability to initiate appropriate gene expression programs. By contrast, *Jmjd2c*-knockout cells remain capable of adopting extra-embryonic endoderm-like phenotypes under permissive conditions. Mechanistically, we show that Jmjd2c is re-distributed to lineage-specific enhancers in primed (serum/LIF) as opposed to naïve (2i/LIF) ESCs. Strikingly, Jmjd2c-bound enhancers are co-occupied by the antagonistic enzyme G9a (also known as Ehmt2), independently of its silencing H3K9-modifying activity. We show that Jmjd2c and G9a co-occupancy coincides with the formation of activating, Med1-containing enhancer complexes. Loss of Jmjd2c abrogates G9a recruitment at Jmjd2c-bound distal sites, which correlates with inefficient loading of mediator-cohesin complexes in ESC-derived EpiSCs and impacts on gene activation upon lineage specification. Collectively, these data reveal that Jmjd2c is required for successful gene transcription and somatic differentiation in pluripotent stem cells, and propose a novel regulatory role for Jmjd2c in stabilising the assembly of essential enhancer-protein complexes at the onset of ESC differentiation.

## RESULTS

### Jmjd2c is required for embryonic stem cell differentiation towards somatic lineages

**Fig. 1.**
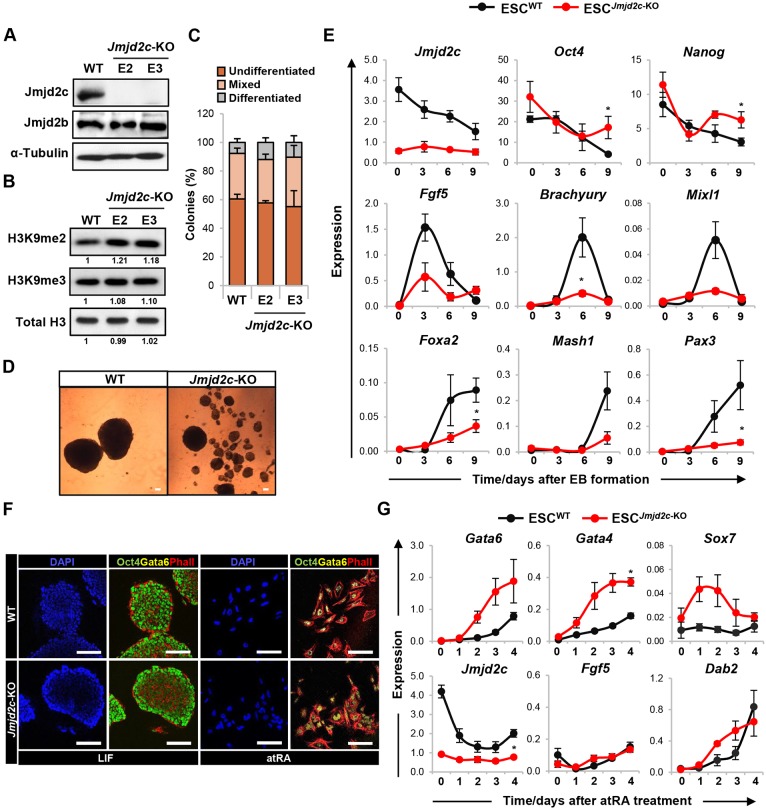
***Jmjd2c*-knockout ESCs can self-renew but fail to differentiate into somatic lineages.** (A) Western blot using anti-Jmjd2c and anti-Jmjd2b antibodies of whole-cell extracts from wild-type (WT) JM8-ESCs and *Jmjd2c*-knockout (*Jmjd2c*-KO) cell lines (E2 and E3). α-Tubulin is used as loading control. (B) Western blots showing bulk levels of H3K9me2, H3K9me3 and total histone H3 in acid-extracted histone lysates from wild-type and *Jmjd2c*-KO cells. Signal quantification is presented relative to wild type. (C) Ability of wild-type and *Jmjd2c*-KO cells to self-renew. Cells were plated at low density and cultured for 5 days with LIF. Colonies were scored as undifferentiated, mixed or differentiated based on alkaline phosphatase activity. Data represent mean±s.e.m. of four experiments. (D) Phase-contrast images of day 9 embryoid bodies (EBs) formed from wild-type and *Jmjd2c*-KO (E3) ESCs. Scale bars: 100 µm. (E) Expression profiling of *Jmjd2c*, pluripotency-associated (*Nanog*, *Oct4*), epiblast (*Fgf5*), mesoderm (brachyury, *Mixl1*), endoderm (*Foxa2*) and neuroectoderm (*Mash1*, *Pax3*) markers during EB-mediated differentiation, as assessed by RT-qPCR and normalised to housekeeping genes. Data represent mean±s.e.m. of at least three experiments. *P<0.05; Mann–Whitney *U*-test at peak time-points. (F) Immunofluorescence staining for Oct4 (green), Gata6 (yellow) and phalloidin (red) in wild-type and *Jmjd2c*-KO (E3) ESCs maintained under proliferative conditions or upon 1 µM retinoic acid (atRA) addition and LIF removal for 4 days. Scale bars: 100 µm. (G) Transcript levels of *Gata6*, *Gata4*, *Sox7* and *Dab2* (PrE markers), *Jmjd2c* and *Fgf5*, as assessed during atRA-induced differentiation. Expression is normalised to housekeeping genes and data show mean±s.e.m. of three experiments. *P<0.05; Mann–Whitney *U*-test at day 4.

Combining genetic deletion and functional assays, we tested whether Jmjd2c plays a role at the exit of pluripotency. *Jmjd2c* mutant and wild-type JM8-ESCs were obtained through the EUCOMM/IKMC repository (Bradley et al., 2012; Skarnes et al., 2011), and targeting of both *Jmjd2c* alleles in *Jmjd2c*-knockout (E2 and E3) ESC clones was confirmed by long-range PCR and GFP expression (Fig. S1A-C). Western blotting validated that full-length Jmjd2c protein expression was completely abolished in *Jmjd2c*-knockout samples (Fig. 1A). This was accompanied by a notable increase in bulk H3K9me2 levels relative to wild type (Fig. 1B, Fig. S1D), an effect attributed to the loss of Jmjd2c itself in the continued expression of other H3K9 demethylases and methyltransferases (Fig. S1D,E). Constitutively *Jmjd2c*-depleted E2 and E3 ESC clones grew normally in medium supplemented with serum plus LIF (Fig. S1F), in agreement with a similar conditional knockout model (Pedersen et al., 2014). When plated at low density, these cells also displayed comparable proportion of undifferentiated colonies relative to their wild-type counterparts (Fig. 1C), despite reduced expression levels of pluripotency-associated factors (Fig. S1G). *Jmjd2c* depletion did not, however, result in general de-repression of differentiation-associated genes (Fig. S1H), including those co-bound by Jmjd2c and PRC complexes at their promoter regions (Das et al., 2014). These data indicate that constitutive *Jmjd2c*-knockout ESCs retain a self-renewing and undifferentiated phenotype in pluripotency culture conditions.

To assess the effect of *Jmjd2c* deficiency on differentiation, *Jmjd2c*-knockout and wild-type ESCs were induced to form embryoid bodies (EBs) (Fig. 1D,E). In contrast to the dispensability of Jmjd2c for ESC self-renewal, *Jmjd2c-*deficient cells were unable to properly differentiate into derivatives of the three germ layers. These cells formed smaller EBs (Fig. 1D) that, in contrast to wild-type EBs, showed residual expression of the pluripotency factors *Oct4* and *Nanog* (Fig. 1E, upper panel). Notably, *Jmjd2c*-knockout EBs showed impaired expression of the epiblast marker *Fgf5* and several mesoderm, endoderm and ectoderm germ layer-affiliated genes (Fig. 1E, lower panels). Importantly, this defect was recapitulated using another loss-of-function strategy (Fig. S2). Using validated puromycin-selectable shRNA vectors (Loh et al., 2007), we could indeed stably establish several *Jmjd2c*-knockdown E14-ESC clones (Fig. S2A-C). As *Jmjd2c*-knockout ESCs, these clones proliferated normally, showing no incidence of spontaneous differentiation and/or prominent gene de-repression (Fig. S2D,E). Moreover, we found that *Jmjd2c*-knockdown ESCs failed to potently activate brachyury and *Mixl1* upon EB formation, despite evidence for *Fgf5* induction at variable levels in different clones and experiments (Fig. S2F; data not shown). Collectively, these findings demonstrate that Jmjd2c is important for the successful differentiation of ESCs into multiple somatic lineages.

Strikingly, however, *Jmjd2c*-deficient cells could differentiate upon LIF withdrawal and addition of all-trans retinoic acid (atRA), indicating that not all differentiation pathways in ESCs were compromised in the absence of Jmjd2c. Differentiation was evidenced by a complete loss of Oct4 expression (Fig. 1F), and prominent upregulation of *Gata6*, *Gata4*, *Sox7* and *Dab2* transcripts (Fig. 1G), consistent with the preferential acquisition of a primitive endoderm (PrE)-like phenotype under these conditions (Artus et al., 2010; Capo-Chichi et al., 2005). In atRA-treated wild-type ESCs, PrE-like differentiation was marked by the swift downregulation of *Jmjd2c* (Fig. 1G, bottom panel), similar to what was observed upon trophoblast lineage commitment *in vitro* (Alder et al., 2010). These findings corroborate with *in vivo* studies showing that *Jmjd2c* expression is dynamically lost in the primitive endoderm, while being retained in the epiblast of peri-implantation embryos (Fig. S3) (Boroviak et al., 2015; Burton et al., 2013). Hence, Jmjd2c might be expendable for the formation of extra-embryonic lineages where it is not normally expressed, an observation that was substantiated by the successful generation of extra-embryonic endoderm (XEN) stem cells from *Jmjd2c*-knockout ESCs (Fig. S4) (Cho et al., 2012; Kunath et al., 2005; Niakan et al., 2013). Collectively, our results support a selective requirement for Jmjd2c during epiblast-derived lineage specification.

### Lack of transcriptional gene priming and skewed cell fate in Jmjd2c-deficient epiblast stem cells

To explore the timing of Jmjd2c function in somatic differentiation, we first examined whether Jmjd2c was required during the transition from ESC to EpiSC pluripotent states (Guo et al., 2009). Both *Jmjd2c*-knockout and wild-type EpiSC lines could be stably established (Fig. S5). However, during this process, we noticed a delay in the induction of the early EpiSC markers *Otx2* and *Dnmt3b* (Tesar et al., 2007; Veillard et al., 2014) in *Jmjd2c*-knockout relative to wild-type ESCs when treated with activin and fibroblast growth factor (Fig. S5A). Notably, lower *Fgf5* mRNA levels were detected in stably converted EpiSCs (cEpiSCs) in the absence of Jmjd2c. Hence, although *Jmjd2c*-deficient ESCs retain the ability to convert into EpiSCs, these cells harbour an incomplete/immature epiblast-like state, which is further suggested by a lack of low-level transcripts at primed germ layer (brachyury and *Foxa2*) markers in *Jmjd2c*-knockout cEpiSCs (Fig. S5A, lower panel).

**Fig. 2.**
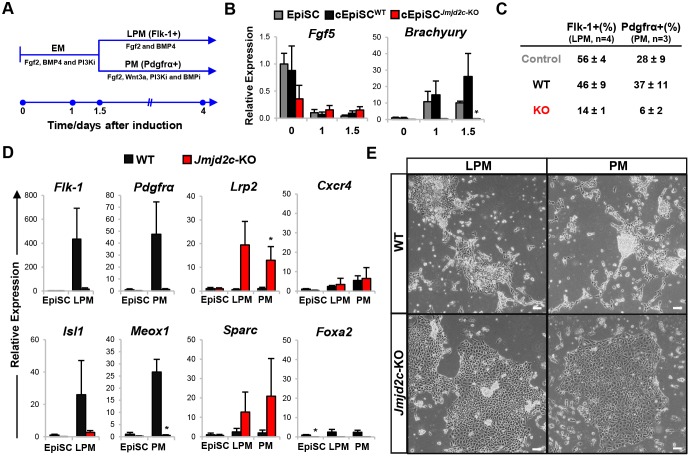
**Skewed differentiation of *Jmjd2c*-knockout cEpiSCs upon mesoderm induction.** (A) Timeline of EpiSC induction towards early mesodermal (EM) progenitors and mature lateral plate mesoderm (LPM) and paraxial mesoderm (PM) cells. (B) Expression levels of *Fgf5* and brachyury in control embryo-derived EpiSCs (grey), wild-type (black) and *Jmjd2c*-KO (red) cEpiSCs upon EM induction. Data are normalised to housekeeping genes and expressed relative to control EpiSCs as mean±s.e.m. of three experiments. *P<0.05; Mann–Whitney *U*-test. (C) Average percentage (±s.e.m.) of Flk1-positive LPM and Pdgfrα-positive PM differentiated cells, as monitored by flow cytometry in control, wild-type and *Jmjd2c*-KO cultures at day 4 post-induction in at least three experiments. (D) Expression of lateral plate (*Kdr*/*Flk1* and *Isl1*) and paraxial (*Pdgfra* and *Meox1*) mesoderm; extra-embryonic endoderm (*Lrp2* and *Sparc*); and definitive (*Cxcr4*) and pan (*Foxa2*) endoderm markers in wild-type and *Jmjd2c*-KO differentiated cells under LPM/PM conditions. Data are normalised to housekeeping genes and expressed relative to wild type (day 0) as mean±s.e.m. of three experiments. *P<0.05; Mann–Whitney *U*-test. (E) Phase-contrast images of LPM and PM wild-type and *Jmjd2c*-KO cultures. Scale bars: 100 µm.

To determine whether *Jmjd2c* deficiency also impacts on the ability of cEpiSCs to respond to differentiation cues, we compared the behaviour of *Jmjd2c*-knockout and wild-type cEpiSCs when prompted to differentiate towards mesodermal lineages. Here, we used a protocol adapted from a human ESC differentiation model (Cheung et al., 2012) to induce early mesodermal (EM) progenitors and mature lateral plate mesodermal (LPM) and paraxial mesodermal (PM) cell types (Fig. 2A). Both control EpiSCs and wild-type cEpiSCs readily acquired an EM-like identity, as typified by the loss of *Fgf5* expression and acquisition of the early primitive streak marker brachyury at day 1-1.5 post-induction (Fig. 2B). By contrast, we found that *Jmjd2c*-knockout cEpiSCs were unable to efficiently progress into early, brachyury-positive, mesodermal progenitors, confirming that differentiation blockage occurs at an early epiblast-like stage in the absence of Jmjd2c.

All three cell lines were further differentiated under LPM and PM conditions, generating Flk1- and Pdgfrα-expressing cell populations, respectively, as monitored by flow cytometry on day 4 post-induction (Fig. 2C). Although the wild-type cEpiSC response closely mirrored that of embryo-derived EpiSCs, differentiation into mature cell types was blocked in *Jmjd2c-*knockout cultures, showing impaired expression of LPM (*Kdr*/*Flk1* and *Isl1*) and PM (*Pdgfra* and *Meox1*) markers. Instead, and irrespective of LPM and PM conditions, *Jmjd2c*-knockout cells induced typical post-implantation extra-embryonic endoderm (*Lrp2* and *Sparc*) but not definitive and pan endodermal (*Cxcr4* and *Foxa2*) markers (Fig. 2D, right panels). Moreover, these cells acquired an adhesive, polarised epithelium-like morphology (Fig. 2E) and stained positive for E-cadherin (Fig. S6) similar to what is observed upon BMP4-induced differentiation of XEN cells (Artus et al., 2012; Paca et al., 2012). Collectively, our findings suggest that, in the absence of Jmjd2c, epiblast cell fate might default towards extra-embryonic endoderm-like derivatives, as tested here upon mesodermal lineage induction.

### *De novo* Jmjd2c recruitment to H3K4me1/me2-rich lineage-specific enhancers in primed embryonic stem cells

We have shown that *Jmjd2c* loss in ESCs is sufficient to inhibit somatic differentiation, despite *Jmjd2c* being normally downregulated upon induction (Fig. 1E, upper panel). This suggests an important role for Jmjd2c in undifferentiated ESCs either in preserving their multi-lineage potential or in mediating the transition from pluripotent ESC to differentiated states. To address the molecular basis for the skewed differentiation of *Jmjd2c*-knockout ESCs, we mapped and compared Jmjd2c DNA-binding sites by ChIP-seq in naïve (2i/LIF) and primed (serum/LIF) ESCs (Marks et al., 2012). To achieve this, E14-ESC clones stably expressing Flag-tagged Jmjd2c were generated (ESC^FV-Jmjd2c-WT^, Fig. S7), and expanded in either condition prior to anti-Flag ChIP (Fig. S8A,B).

**Fig. 3.**
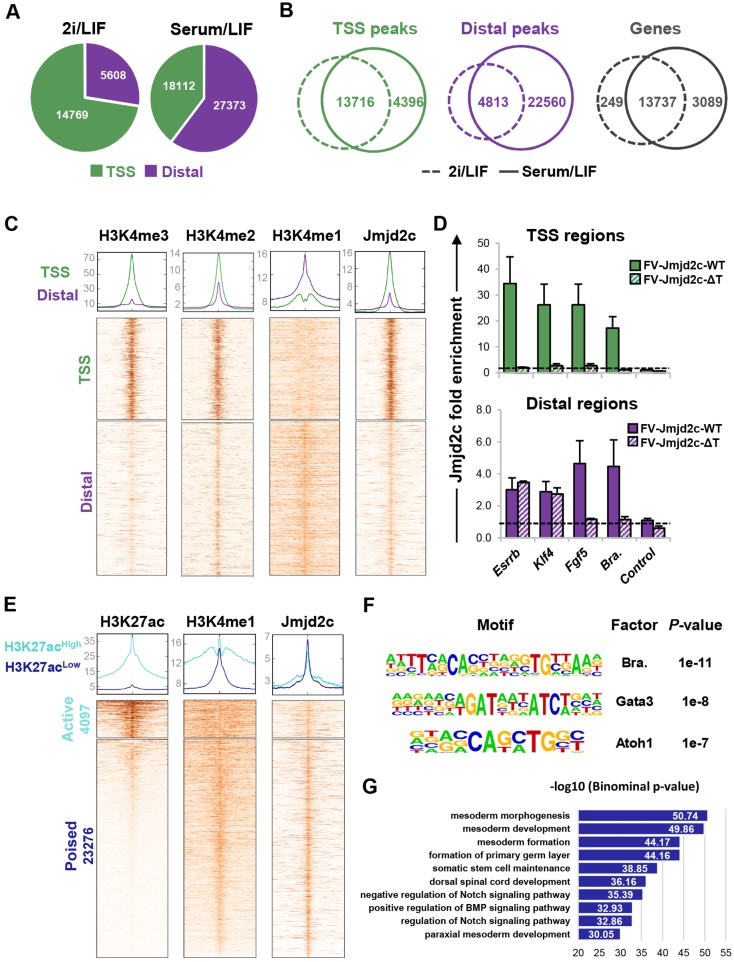
**Jmjd2c preferentially overlaps with H3K4me1/me2-rich lineage-specific enhancers in primed ESCs.** (A) Numbers of Jmjd2c peaks located within 1 kb of transcriptional start sites (TSS) or outside this interval (distal) in 2i/LIF and serum/LIF. (B) Venn diagrams indicating the overlap of Jmjd2c-bound TSS and distal peaks in serum/LIF with at least one peak in 2i/LIF (minimum overlap=1 bp); overlap of the nearest gene to Jmjd2c peaks in the two conditions (right panel). (C) Density heatmaps of H3K4me3/2/1 and Jmjd2c levels across a 10 kb window centred at TSS and distal Jmjd2c-bound regions in serum/LIF. (D) Enrichment levels for full-length Jmjd2c (FV-Jmjd2c-WT) and a mutant form of Jmjd2c lacking Tudor domains (FV-Jmjd2c-ΔT) at TSS and enhancer sites of active (*Esrrb* and *Klf4*) and lineage-specific (*Fgf5* and brachyury) genes in ESCs. Fold enrichment is relative to control cells (empty vector; dotted line). Data represent mean±s.e.m. of three experiments. Background level is confirmed at an intergenic region. (E) Average density plots and heatmaps of H3K27ac, H3K4me1 and Jmjd2c levels across a 10 kb window centred at Jmjd2c-bound distal sites in serum/LIF. Peaks are sorted according to H3K27ac levels using a *k*-means clustering algorithm. (F) Motifs identified among overrepresented binding sequences at H3K27ac-low Jmjd2c distal peaks. (G) Top 10 most significant biological functions of genes associated with H3K27ac-low Jmjd2c distal peaks.

In 2i/LIF, over 70% of Jmjd2c peaks were detected within 1 kb of the transcriptional start sites (TSS) (Fig. 3A), as previously reported (Pedersen et al., 2014). Strikingly, however, Jmjd2c genomic distribution was altered during the priming of ESCs for differentiation. Substantially more Jmjd2c peaks (45,485 versus 20,377; FDR<0.0001) were detected in serum/LIF relative to 2i/LIF conditions. Moreover, we found that the majority of the peaks specific to the serum/LIF state were located more than 1 kb away from the TSS interval, hereafter referred to as distal peaks (Fig. 3A; Fig. S8C). This trend was recapitulated by interrogating an independent ChIP-seq dataset (Fig. S8D) (Das et al., 2014). Importantly, we identified that the sets of genes targeted by Jmjd2c in the two ESC states were largely overlapping, as revealed using gene annotation analysis (Fig. 3B). Hence, these findings uncover that, in 2i/LIF, Jmjd2c primarily binds to TSS regions, yet additionally occupies distal regions affiliated with the same cohort of genes in serum/LIF, as further validated at selected (*Fgf5* and brachyury) loci by ChIP-qPCR on endogenous Jmjd2c protein in naïve (2i/LIF) and primed (serum/LIF) wild-type and *Jmjd2c*-knockout ESCs (Fig. S8E-G).

Jmjd2c-bound distal peaks most closely overlapped with H3K4me1-rich sites (Fig. 3C), which typically define enhancers (Creyghton et al., 2010; Zentner et al., 2011). As expected, Jmjd2c-bound TSS regions were preferentially decorated with H3K4me3, whereas H3K4me2 could be detected at both TSS and distal peaks. Given that Jmjd2c can bind both H3K4me2 and H3K4me3 in a Tudor domain-dependent manner (Pedersen et al., 2014), we asked whether Jmjd2c recruitment at enhancers might be at least partly mediated via H3K4me2 recognition. Combining mutagenesis and ChIP-qPCR analyses, we showed that Jmjd2c binding was abrogated at TSS and distal regions in ESCs expressing a mutant version of Jmjd2c lacking its two Tudor domains (ESCs^FV-Jmjd2c-ΔT^) (Fig. 3D; Fig. S7D), indirectly validating H3K4-dependent Jmjd2c DNA binding. Strikingly, however, FV-Jmjd2c-ΔT binding was selectively retained at ESC-specific (*Esrrb* and *Klf4*) enhancers (Fig. 3D, bottom panel), suggesting a different mode of Jmjd2c recruitment at these sites possibly via cooperation with additional enhancer-bound proteins, including Jmjd2b and Nanog, as previously suggested (Das et al., 2014).

Active and poised enhancers can be distinguished genome-wide by the presence or absence of p300-mediated H3K27ac marks (Creyghton et al., 2010; Zentner et al., 2011). Interestingly, we found that 85% of Jmjd2c-bound distal peaks harboured low or no H3K27ac deposition (Fig. 3E), indicating that Jmjd2c is prevalently recruited to poised enhancers in serum/LIF. Known motif sequence enrichment analysis at these sites disclosed a high incidence of motifs for differentiation-associated factors, such as brachyury, Gata3 and Atoh1 (Fig. 3F), typifying tissue-specific enhancers activated later on during development. Concordantly, Gene Ontology analysis revealed significant enrichment for developmental and somatic differentiation processes at Jmjd2c-bound, H3K27ac-low sites (Fig. 3G). These findings support a role for Jmjd2c in promoting differentiation into derivatives of the three primary germ layers. Taken together, our findings reveal that Jmjd2c is recruited in a timely manner to lineage-specific enhancers during ESC priming for differentiation.

### The antagonistic enzymes Jmjd2c and G9a are co-enriched at active and poised enhancers independently of H3K9-modifying activities

Consistent with its H3K9-demethylase activity (Cloos et al., 2006; Whetstine et al., 2006), we showed that constitutively depleting Jmjd2c in ESCs leads to a noticeable increase in bulk H3K9me2 levels (Fig. 1B, Fig. S1D). Deposition of H3K9 and DNA methylation are known mechanisms operating in extra-embryonic tissues and derived stem cells to prevent the expression of somatic, lineage-specific genes (Alder et al., 2010; Senner et al., 2012). In light of the skewed differentiation of *Jmjd2c*-knockout ESCs towards extra-embryonic fates, we thus asked whether Jmjd2c occupancy might antagonise the acquisition of repressive marks in pluripotent stem cells.

**Fig. 4.**
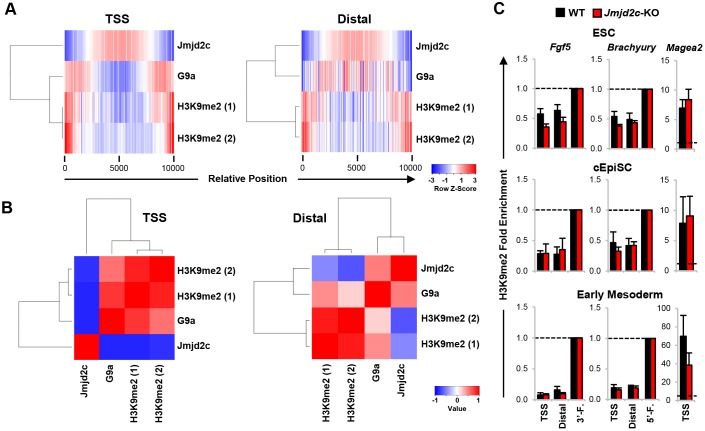
**G9a is enriched at distal Jmjd2c-bound sites independently of its H3K9-modifying activity.** (A) Heatmap distributions (i.e. binned mean ChIP-seq read density) of Jmjd2c, G9a (Mozzetta et al., 2014), H3K9me2 (1) (Liu et al., 2015) and H3K9me2 (2) (Das et al., 2014) across Jmjd2c-bound TSS and distal sites. Each ChIP-seq experiment is adjusted for sequencing depth and normalised to respective input. Colour key indicates enrichment levels from low to high. (B) Heatmap showing Pearson correlation between Jmjd2c, G9a and H3K9me2 distributions across Jmjd2c-bound TSS and distal sites. Colour key indicates correlation coefficient from low to high. (C) Enrichment levels for H3K9me2 at TSS, distal/enhancer and Jmjd2c-free flanking regions of *Fgf5* and brachyury, as determined by ChIP-qPCR in wild-type and *Jmjd2c*-KO ESCs and cEpiSCs, and upon cEpiSC mesodermal induction. Data are relative to flanking regions (dotted line) and are shown as mean±s.e.m. of three experiments. *Magea2* TSS is used as positive control.

At the chromosomal level, G9a-mediated H3K9me2 deposition encompasses large domains yet is depleted at the TSS regions of active and lineage-specific genes in ESCs (Lienert et al., 2011). We confirmed genome-wide that Jmjd2c and H3K9me2 are mutually exclusive across Jmjd2c-bound TSS regions, whereas G9a and H3K9me2 are confined to regions flanking Jmjd2c peak summits, as demonstrated using heatmap distributions and Pearson's correlation coefficients (Fig. 4A,B, left panels). In striking contrast, however, Jmjd2c and G9a largely overlapped across enhancer regions in the absence of H3K9me2 deposition (Fig. 4A,B, right panels), as observed at both active and poised Jmjd2c-bound distal sites (Fig. S9). The colocalisation of the two antagonistic enzymes Jmjd2c and G9a at enhancers suggests a possible counteracting mechanism in the control of gene expression. Loss of Jmjd2c, however, did not lead to increased levels of H3K9me2 and *de novo* DNA methylation (Fig. 4C; data not shown), as examined at the locus level across *Fgf5* and brachyury regulatory regions in *Jmjd2c*-knockout ESCs, and in derived cEpiSCs and induced-mesodermal progenitors where *Fgf5* and brachyury activation is respectively inhibited in the absence of Jmjd2c (Fig. 2). These data strongly suggest that, in contrast to what we hypothesised, the failure of *Jmjd2c*-knockout cells to differentiate towards somatic derivatives is not due to a lack of Jmjd2c-mediated protection against the acquisition of repressive marks at Jmjd2c-bound gene targets.

**Fig. 5.**
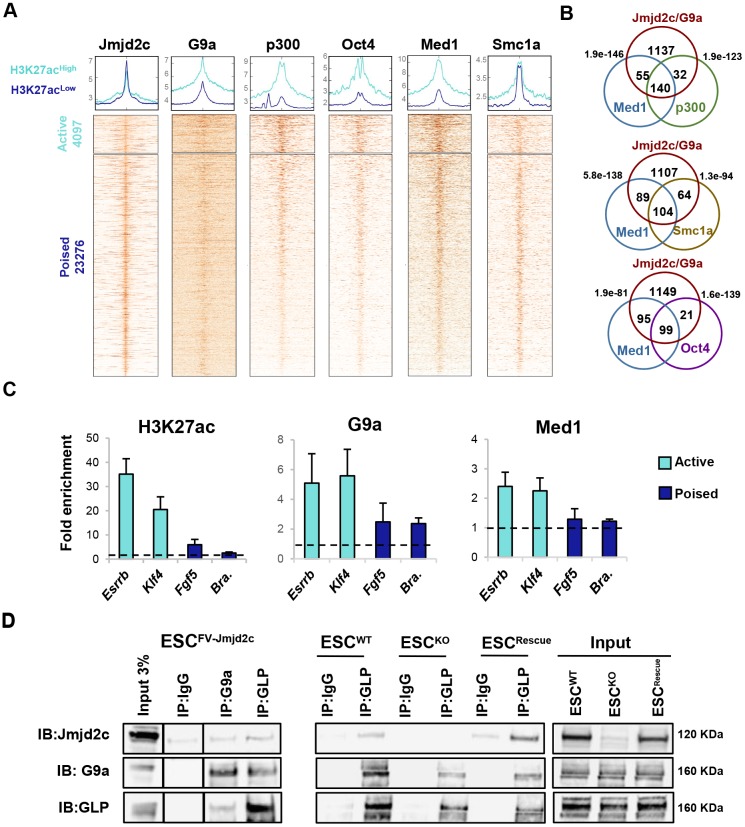
**Jmjd2c and G9a co-occupy active and poised enhancers in ESCs.** (A) Average density plots and heatmaps showing enrichment levels for Jmjd2c, G9a, p300, Oct4, Med1 and Smc1a within a 10 kb window centred at H3K27ac-high (light blue) and H3K27ac-low (dark blue) Jmjd2c distal peaks. Scales are adjusted to maximum peak intensity for each dataset. (B) G9a peaks intersecting with Jmjd2c-distal H3K27ac-high sites are selected for this analysis. Depicted are the numbers of G9a/Jmjd2c peaks overlapping with Med1/p300, Med1/Oct4 and Med1/Smc1a (maximum gap 200 bp). *P*-values are calculated using hypergeometric tests. (C) Enrichment levels for H3K27ac, G9a and Med1 at enhancers of active (*Esrrb* and *Klf4*) and lineage-specific (*Fgf5* and brachyury) genes, as assessed by ChIP-qPCR. Data are relative to an intergenic region (dotted line) and represent mean±s.e.m. of three experiments. (D) (Left) G9a and GLP immunoprecipitations performed in nuclear fractions of ESCs expressing Flag-tagged Jmjd2c (FV-Jmjd2c). Input corresponds to control ESCs. Lanes were cropped and repositioned for clarity. (Middle and right panels) GLP immunoprecipitation performed in nuclear fractions of wild-type, *Jmjd2c*-KO and full-length *Jmjd2c* rescue ESCs. Interactions are visualised using immunoblotting. Data represent duplicate experiments.

Surprisingly, we found that the highest levels of G9a were detected at active (H3K27ac-high) Jmjd2c-bound distal sites (Fig. S10A, left panel), as exemplified at *Esrrb* and *Klf4* loci (Fig. S10B,C). Moreover, G9a significantly overlapped with known enhancer-associated factors, including p300, Oct4 and the subunits of mediator (Med1) and cohesin (Smc1a) complexes at these sites in ESCs (Fig. 5A,B). Lower yet detectable G9a enrichment, with peaks closely aligned with Jmjd2c, p300, Oct4, Med1 and Smc1a, also marked poised (H3K27ac-low) Jmjd2c-bound enhancers (Fig. 5A, lower panels), as illustrated at *Fgf5* and brachyury loci (Fig. S10D-E). The detection of differential G9a enrichment levels at ESC-specific and transcriptionally primed loci was furthermore verified by ChIP-qPCR in JM8-ESCs (Fig. 5C), and validated in control and *G9a*-knockout ESCs, where G9a precipitation was abolished, as expected (Fig. S11A-C). Interestingly, although Jmjd2c binding remained unaltered in *G9a*-knockout ESCs, G9a was lost at *Fgf5* and brachyury enhancers in the absence of Jmjd2c (Fig. S11D), suggesting a possible role for Jmjd2c in facilitating G9a binding. Jmjd2c-G9a co-occupancy was furthermore supported by the identification of a physical interaction between Jmjd2c and G9a/GLP (Fig. 5D), and their simultaneous detection at the same enhancer fragments in sequential ChIP (re-ChIP) assays (Fig. S11E). Importantly, Jmjd2c and G9a also interact with Med1 (Fig. S11F-G), further implying that Jmjd2c-G9a co-occupancy might coincide with the assembly of activating protein mega-complexes at enhancers. Taken together, our findings unveil Jmjd2c and G9a as novel enhancer-associated factors, and demonstrate that these molecules are co-recruited genome-wide to active and poised enhancers in ESCs independently of their H3K9-modifying activities.

### Jmjd2c facilitates the assembly of essential enhancer-protein complexes in ESC-derived epiblast stem cells

**Fig. 6.**
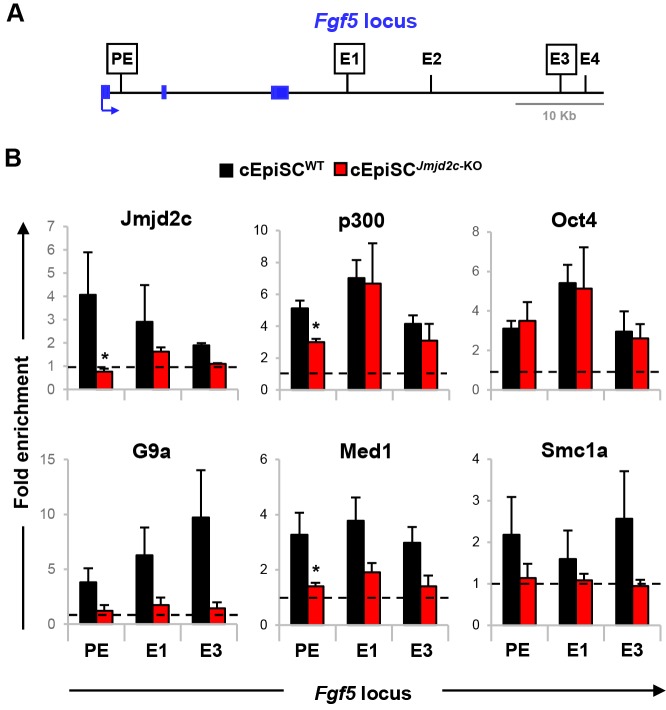
**Assembly of enhancer-protein complexes is destabilised in the absence of Jmjd2c in ESC-derived EpiSCs.** (A) Mapping of poised (PE) and epiblast-specific enhancer sites (E1-E4) at *Fgf5* locus (Buecker et al., 2014). (B) Enrichment levels for Jmjd2c, p300, Oct4, G9a, Med1 and Smc1a at indicated sites as assessed by ChIP-qPCR in wild-type and *Jmjd2c*-KO cEpiSCs. Fold enrichment is relative to an intergenic region (dotted line) and represents mean±s.e.m. of three experiments. *P<0.05; Mann–Whitney *U*-test.

We observed that, similar to H3K4me1, Jmjd2c pre-marks a large panel of tissue-specific enhancers in ESCs prior to gene activation and lineage specification (Fig. 3). We therefore hypothesised that, instead of protecting regulatory regions from the acquisition of repressive marks, Jmjd2c might facilitate and/or stabilise the assembly of enhancer-associated proteins, including G9a, at the onset of differentiation. To explore this, we compared the enrichment profiles of Jmjd2c, p300, Oct4, G9a, Med1 and Smc1a in *Jmjd2c*-knockout and wild-type ESC-derived cEpiSCs by ChIP-qPCR. For this analysis, we focussed on the previously delineated *Fgf5* poised enhancer (PE) in ESCs, or on newly established (E1 and E3) enhancers produced by *Fgf5* activation in post-implantation epiblast-like cells (Fig. 6A) (Buecker et al., 2014). These sites are co-occupied by Jmjd2c and G9a, as verified in wild-type cEpiSCs (Fig. 6B, left panel).

All three *Fgf5* enhancer (PE, E1 and E3) elements examined were similarly bound by Oct4 and p300, and harboured high levels of p300-mediated H3K27ac deposition in wild-type and *Jmjd2c*-knockout cEpiSCs (Fig. 6B; data not shown). This suggests that Jmjd2c is not required for the binding of the pioneer transcription factor Oct4 and/or for the establishment of permissive chromatin at enhancers. Despite similar detection of protein levels in wild-type and knockout samples (Fig. S12A), we observed that Jmjd2c loss abrogated G9a recruitment, as seen at the ESC stage (Fig. S11D, right panel), and furthermore destabilised the loading of the essential enhancer-associated factors Med1 and Smc1a (Fig. 6B), potentially impeding *Fgf5* expression in *Jmjd2c*-knockout cEpiSCs (Fig. S5A). Remarkably, we identified that G9a, Med1 and Smc1a binding were similarly compromised at poised enhancers of germ layer markers (*Zic1* and brachyury) prior to gene activation (Fig. S12B). Based on these findings, we propose that inefficient assembly of activating protein complexes at poised and newly established enhancers could account for the impaired activation of lineage-affiliated genes that we have observed upon somatic differentiation in *Jmjd2c*-knockout pluripotent stem cells (Figs 1E and 2).

## DISCUSSION

In this study, we have identified a novel regulatory function for Jmjd2c at tissue-specific enhancers during ESC priming for differentiation. We show that Jmjd2c is required for successful multi-lineage differentiation, as assessed by EB formation. In the absence of Jmjd2c, EBs were smaller and ineffective at inducing expression of differentiation-associated genes, including early markers of the three mesoderm, endoderm and ectoderm lineages. Moreover, we established that ESC differentiation was impeded or stalled at an early post-implantation epiblast-like stage. Indeed, while *Jmjd2c*-knockout ESCs could transit into self-renewing cEpiSCs, these cells failed to establish a functionally primed state – a point that was reinforced here by their inability to further progress into mesodermal progenitors. By contrast, we found that *Jmjd2c*-knockout ESCs could readily differentiate into primitive endoderm-like derivatives under permissive conditions, as recently confirmed in triple *Jmjd2abc*-knockout models (Pedersen et al., 2016). Interestingly, however, among the three *Jmjd2* family members, *Jmjd2c* is uniquely downregulated in the primitive endoderm of the developing blastocyst, while being upregulated in the epiblast at peri-implantation times (Fig. S3) (Boroviak et al., 2015). This *in vivo* expression pattern most closely concurs with a primary role for Jmjd2c in the epiblast, while being dispensable for the formation of extra-embryonic tissues, as demonstrated in this study using ESC, EpiSC and XEN *in vitro* models.

We confirmed that *Jmjd2c* knockout is not detrimental to ESC proliferation and the maintenance of an undifferentiated phenotype upon prolonged culture (Pedersen et al., 2014), a conclusion here extended to ESC-derived EpiSCs. These results reiterate the robustness of pluripotent stem cell self-renewal, most likely reflecting compensation mechanisms among related transcriptional regulators, including Jmjd2 family members (Pedersen et al., 2016). These results, however, contrast with earlier studies showing that shRNA-mediated *Jmjd2c* depletion led to spontaneous ESC differentiation and/or de-repression of many lineage-specific markers (Das et al., 2014; Loh et al., 2007). The discrepancies between these differing phenotypes could relate to differences in genetic backgrounds, culture conditions and the use of different knockdown constructs versus the study of constitutive knockout ESC models. Importantly, our conclusion that *Jmjd2c*-depleted ESCs self-renew normally yet fail to properly differentiate into epiblast-derived progenitors is based on concurring functional characterisation of *Jmjd2c*-knockout and -knockdown models, thus ruling out off-target effects commonly associated with shRNA approaches. Previously published studies of Jmjd2c function in ESCs (Das et al., 2014; Loh et al., 2007; Pedersen et al., 2014) did not investigate the impact of *Jmjd2c* depletion on the differentiation abilities of ESCs upon EB formation and lineage-specific induction, which precludes further comparison.

Interestingly, ectopically expressing *Jmjd2c* in ESCs also led to differentiation inhibition upon EB formation (data not shown). This finding, together with Jmjd2c being normally downregulated during this process, points to an important role for this protein at the onset of differentiation. Accordingly, we found that Jmjd2c is recruited in a timely manner to lineage-specific enhancers upon ESC priming for differentiation. By directly comparing Jmjd2c genome-wide DNA-binding sites in 2i/LIF and serum/LIF, we confirmed that Jmjd2c is prevalently bound to H3K4me3-rich TSS regions of active and differentiation-associated genes in 2i/LIF. Strikingly, however, we identified that a significant fraction of serum/LIF-specific Jmjd2c-binding sites maps away from TSS regions, overlapping with H3K4me1/me2-rich enhancer regions in the vicinity of the same cohort of gene targets. Although Jmjd2c binding is less abundant at distal relative to TSS sites (Fig. S8 and Fig. S10), we confirmed that Jmjd2c is similarly recruited, at least in part, to enhancers and cognate promoters via its Tudor domains, most likely by recognition of H3K4me2/1 and H3K4me3, respectively (Pedersen et al., 2014). This corroborates with Jmjd2c detection in H3K4me3- and H3K4me1-coupled proteome datasets, as recently reported (Engelen et al., 2015). Given that regulatory regions nucleate the binding of numerous transcriptional regulators and co-factors, resident molecules might also contribute to Jmjd2c recruitment via protein-protein interaction at these sites.

Remarkably, however, Jmjd2c was found similarly enriched at active and pre-marked (poised) enhancers in serum/LIF (Fig. 5), suggesting that Jmjd2c could act as a molecular platform for the recruitment of enhancer constituents. In particular, we asked whether the loss of Jmjd2c in ESCs might impact on Oct4 and p300 re-distribution at newly activated (*Fgf5*) enhancer sites upon ESC-to-EpiSC conversion. Binding of Oct4, p300 and p300-mediated H3K27ac deposition were, however, retained at *Fgf5* in *Jmjd2c*-knockout cEpiSCs. Instead, we established that Jmjd2c was required for the proper binding of Med1 and Smc1a at active (*Fgf5*) and poised enhancers in epiblast-like cells (Fig. 6 and Fig. S12). A facilitating role for Jmjd2c in the assembly of activating enhancer-protein complexes was also supported by the ability of Jmjd2c to physically interact with G9a and Med1 in ESCs. Together, these findings suggest that, in the absence of Jmjd2c, inefficient loading of essential mediator-cohesin complexes at lineage-specific enhancers impairs the activation of affiliated genes, accounting for the differentiation defect observed in *Jmjd2c*-knockout cells.

**Fig. 7.**
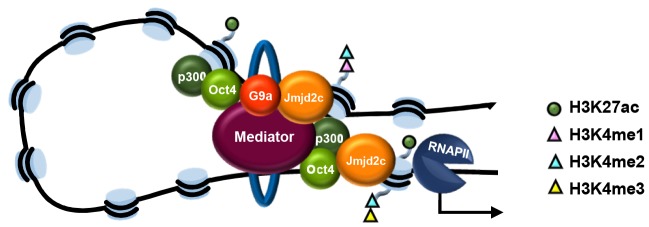
**Proposed model: assembly of activating Jmjd2c-G9a centred enhancer-protein complexes.** Jmjd2c and G9a co-occupy poised (lineage-specific) enhancers in primed ESCs where they stabilise the assembly of mediator-cohesin complexes (Kagey et al., 2010) that are necessary for the formation of DNA loops and for potent gene activation at the exit of pluripotency and upon differentiation.

Given the established role of mediator and cohesin in bridging enhancers and core promoters (Kagey et al., 2010), we speculate that Jmjd2c recruitment to enhancers might coincide and/or contribute to DNA looping events prior to gene activation and lineage specification (Fig. 7). In line with this model, we find that Jmjd2c-bound distal peaks closely align with promoter-enhancer interaction sites, as shown in the vicinity of *Foxa2* in ESCs (Fig. S13). Interestingly, we note that the stability of all Jmjd2c-bound promoter-enhancer interactions observed across the genome is significantly enhanced in serum/LIF when compared with 2i/LIF (CHiCAGO score analysis, *P-value* 2.446×10^−12^; O.J. and H.G.S., unpublished), correlating with Jmjd2c re-distribution to poised enhancers during ESC priming for differentiation. However, and given that ChIP and Hi-C approaches commonly employ crosslinked chromatin, it remains unclear whether the increased detection of Jmjd2c at enhancers might be the cause or the result of stabilised promoter-enhancer interactions in serum/LIF.

An intriguing finding arising from this study is the co-recruitment of the H3K9-methyltransferase G9a to Jmjd2c-bound distal peaks in ESCs. Contrasting with its canonical role in gene silencing (Feldman et al., 2006; Mozzetta et al., 2014), highest G9a co-enrichment levels were unexpectedly observed at ESC-specific, Jmjd2c-bound enhancers, suggesting that G9a/Jmjd2c co-enrichment might coincide with the formation of activating complexes. Moreover, and similar to Oct4, p300, Med1 and Smc1a, G9a was also present at lineage-specific, Jmjd2c-bound enhancers, although at reduced levels in ESCs. In the absence of Jmjd2c, G9a binding was destabilised, as also demonstrated in ESC-derived cEpiSCs along with Med1 and Smc1a. Interestingly, G9a was found to be capable of interacting with Jmjd2c, Med1 or CDYL, suggesting that G9a can form both activating and repressive complexes in ESCs (Fritsch et al., 2010). This is in agreement with previous reports showing that G9a is co-recruited and interacts with either the co-activator Med1 or the co-repressor Jarid1 in a mutually exclusive manner at β-globin genes during haematopoiesis (Chaturvedi et al., 2012; Shankar et al., 2013). Remarkably, and consistent with a dual role for G9a in regulating gene expression, we find that Jmjd2c-G9a co-bound targets are significantly enriched among differentially expressed genes in *G9a*-knockout ESCs (Mozzetta et al., 2014) and E8.5 embryos (Auclair et al., 2016), showing clear evidence for both gene upregulation and downregulation *in vitro* and *in vivo* (Fig. S14). Notably, genes associated with developmental processes are prevalently downregulated in E8.5 embryos in the absence of G9a as previously documented (Auclair et al., 2016; Mozzetta et al., 2014). However, the mechanisms underlying the action of G9a as repressor and activator in ESCs and in other cellular contexts (Chaturvedi et al., 2012; Shankar et al., 2013) remain unknown.

Despite a global increase in H3K9me2 levels in the absence of Jmjd2c, we did not detect any aberrant acquisition of H3K9 and DNA methylation at Jmjd2c/G9a co-bound lineage-affiliated genes in *Jmjd2c*-knockout ESCs, in cEpiSCs or upon lineage specification (Fig. 4; data not shown). This argued against a role for Jmjd2c in continually removing G9a-mediated H3K9me2 deposition, implying novel histone-independent roles for Jmjd2c and G9a at tissue-specific enhancers. Although the precise molecular interplays between the two molecules need to be fully deciphered, we note that G9a automethylation sites, which anchor the binding of repressive complexes (Ruan et al., 2012), were previously identified as potential targets for Jmjd2c-mediated demethylation (Ponnaluri et al., 2009). Whether demethylation of G9a by Jmjd2c via its catalytic domain is a prerequisite for interaction with activating complexes needs to be studied. More generally, further elucidating how Jmjd2c and other histone demethylases might act as key post-translational regulators to promote the assembly of activating enhancer-protein complexes could indeed provide novel important insights into the regulation of enhancer activity and gene expression in stem cells and development.

## MATERIALS AND METHODS

### Cell culture

Mouse ESCs were cultured as previously described (Alder et al., 2010) with 10% FBS (serum), LIF or adapted to serum-free conditions (N2B27 with 1 μM PD0325901, 3 μM CHIR99021 and LIF), as described previously (Ying et al., 2008). Generation of ESC lines are described in the supplementary Materials and Methods, and primers used for genotyping/cloning are listed in Tables S2 and S3.

ESC differentiation was induced in embryoid bodies (EBs) (ultra-low attachment plates, Corning, in 5% FBS without LIF) or with addition of 1 µM all-trans retinoic-acid (atRA) in 5% FBS without LIF. For mesoderm differentiation, EpiSCs were cultured in FLyB media (bFGF, LY294002 and BMP4) for 36 h followed by either FB40 (bFGF and BMP4) or FLyWLDN (bFGF, LY294002, Wnt3a and LDN193189) media as previously described (Cheung et al., 2012). Full media composition and generation of cEpiSCs are outlined in the supplementary Materials and Methods.

### Quantitative PCR

RNA was isolated with the RNeasy mini kit (Qiagen) and reverse-transcribed using SuperScript II (Invitrogen). cDNA/DNA was amplified with KicQstart SYBR Green PCR Mastermix (Sigma). Details and primer sequences are listed in the supplementary Materials and Methods and Table S4.

### Immunofluorescence staining

Cells fixed with 4% paraformaldehyde were permeabilised/blocked for 30 min in 0.4% TritonX-100 and 10% serum. Primary antibody (see Table S1) incubation occurred overnight at 4°C. Fluorophore-conjugated secondary antibodies (ThermoFisher) were incubated for 1 h. Fluorescence was visualised on a SP5 Leica confocal microscope or a fluorescent/brightfield microscope. For further details, see supplementary Materials and Methods.

### Flow cytometry analysis

Cells were harvested using cell dissociation buffer (Gibco), blocked in 2% FBS and analysed on an Accuri C6 Flow cytometer. For details on the antibodies used, see supplementary Materials and Methods.

### RNA-seq analysis

Computational analysis on previously published RNA-seq datasets generated in G9a-knockout and wild-type embryos and ESCs are described in the supplementary Materials and Methods.

### Immunoblotting, immunoprecipitation, ChIP and ChIP-seq

Whole-cell lysates and acid-extracted histones (Shechter et al., 2007) were resolved on SDS-PAGE gels and transferred into PVDF membranes (GE Healthcare). Immunoprecipitation and chromatin immunoprecipitation (ChIP) were performed as previously described (Battisti et al., 2016; Frank et al., 2001; Fritsch et al., 2010). Experimental details and information about computational analysis are outlined in the supplementary Materials and Methods. Primers used for the ChIP can be found in Tables S5 and S6.
